# The Combination of Temporal and Spatial Dose Fractionation in Microbeam Radiation Therapy

**DOI:** 10.3390/biomedicines13030678

**Published:** 2025-03-10

**Authors:** Jessica Stolz, Kristina Rogal, Sandra Bicher, Johanna Winter, Mabroor Ahmed, Susanne Raulefs, Stephanie E. Combs, Stefan H. Bartzsch, Thomas E. Schmid

**Affiliations:** 1 Department of Radiation Oncology, TUM School of Medicine and Health, Technical University Munich, 81675 Munich, Germany; jessica.stolz@tum.de (J.S.); susanne.raulefs@tum.de (S.R.);; 2 Helmholtz Zentrum München, Institute of Radiation Medicine (IRM), Neuherberg, 85764 Munich, Germany

**Keywords:** microbeam radiation therapy, spatially fractionated radiation therapy, temporal fractionation, lung cancer, CFA

## Abstract

**Background**: Microbeam radiation therapy (MRT) is an advanced preclinical approach in radiotherapy that utilizes spatially fractionated dose distributions by collimating x-rays into micrometer-wide, planar beams. While the benefits of temporal fractionation are well established and widely incorporated into conventional radiotherapy protocols, the interplay between MRT and temporal dose fractionation remains largely unexplored. In this study, we investigate the effects of combining temporal and spatial dose fractionation by assessing clonogenic cell survival following temporally fractionated MRT with varying irradiation angles, compared to conventional broad-beam (BB) irradiation. **Methods**: A lung tumor cell line (A549) and a normal lung cell line (MRC-5) were irradiated with a total number of four fractions with a 24 h interval between each fraction. We compared a temporally fractionated BB regime to two temporally fractionated MRT schemes with either overlapping MRT fields or MRT fields with a 45° rotation per fraction. Subsequently, the clonogenic cell survival assay was used by analyzing the corresponding survival fractions (SFs). **Results**: The clonogenic survival of A549 tumor cells differed significantly between microbeam radiation therapy with rotation (MRT + R) and overlapping MRT. However, neither MRT + R nor overlapping MRT showed statistically significant differences compared to the broad-beam (BB) irradiation for A549. In contrast, the normal tissue cell line MRC-5 exhibited significantly higher clonogenic survival following both MRT + R and overlapping MRT compared to BB. **Conclusions**: This study demonstrates that combining temporal and spatial fractionation enhances normal tissue cell survival while maintaining equivalent tumor cell kill, potentially increasing the therapeutic index. Our findings support the feasibility of delivering temporally fractionated doses using different MRT modalities and provide clear evidence of the therapeutic benefits of temporally fractionated MRT.

## 1. Introduction

Lung cancer is a tremendous global health concern, imposing a substantial social and economic burden on modern society. In 2022, one out of eight newly diagnosed cancers were lung cancer, with an incidence of 2.5 million cases worldwide and a mortality rate of 1.8 million [1]. The staging at the time of diagnosis strongly influences the patient’s outcome. For instance, patients diagnosed with stage I lung cancer have a 5-year survival rate of approximately 60%, whereas those with stage II–IV have varying rates, from 40% to less than 5%. However, 75% of newly diagnosed lung cancer patients are already in the advanced stages of III or IV, leading to a poor prognosis, underscoring the critical importance of early detection and intervention [2].

Today radiotherapy (RT) alone or in combination with surgery, chemotherapy or immunotherapy is a crucial aspect in the treatment of cancer and is administered to over 50% of cancer patients [3]. Furthermore, the clinical goal of RT is to enhance the therapeutic index by administering a tolerable dose, which decreases normal tissue and organs at risk toxicity while increasing tumor control probability as well as exploiting tissue-level recovery processes [4,5]. Almost every radiotherapy is delivered as a temporally fractionated scheme, which enables the repair of sublethal damage between each fraction and enhances healthy cell survival [6]. The biological reasons for fractionated radiotherapy were first summarized by Withers (1975), who coined the term ‘4 R’s of radiobiology’ [7]. The application of temporal fractionation constituted a major advancement in RT, allowing normal tissue to repair sublethal damage between the treatment sessions, in addition to enabling mechanisms of reoxygenation, repopulation and redistribution. Even though dose fractionation results in considerably less severe acute and late side effects compared to a single treatment session, normal tissue toxicity is still considered the main limiting factor in cancer treatment [8].

Despite new developments and milestones in modern medicine such as immunotherapy as a groundbreaking approach, several factors such as tumor staging and the phenotype of the tumor lead to differences in patient outcome [9]. However, 40% of all lung cancer patients undergo external radiotherapy in combination with chemotherapy [10]. Currently, conventional dose fractionation between 1.8 and 2 Gy in daily fractions, five days per week to a total dose of 60 Gy, is prescribed for patients with locally advanced Non-Small Cell Lung Cancer (NSCLC). However, dose escalation is correlated with worse patient overall survival [11].

Another innovative method for achieving an improved therapeutic index in radiotherapy lays within techniques that employ the dose-volume effect, which states the smaller the field size, the higher the tolerance of the healthy tissue [12]. Alban Koehler first investigated the concept of spatial fractionation, at the beginning of the 20th century [13]. Spatially fractionated radiation therapy (SFRT) is an innovative treatment concept, which seeks to exploit dose-volume effects in normal tissues without compromising tumor control. Depending on the field sizes, spatially fractionated radiotherapy can be applied either on a micrometer (microbeam radiation therapy, or MRT), millimeter scale (minibeam radiation therapy, or MBRT) or up to a centimeter by using grid therapy [14].

They achieve dose modulation of the segmentation of the microbeam radiation field with a multi-slit collimator, composed of 8 mm thick blocks of tungsten [15], which results in highly collimated and quasi-parallel 25–100 μm wide arrays of x-ray microbeams separated by center-to-center distances of 200–400 µm [13,16]. Using a collimator, arrays of planar beams that are a few tens of micrometers wide are created, with unconventionally high peak doses, which are separated by low-dose regions (valleys) with doses below the tissue tolerance level [17]. Peak doses during MRT can reach up to thousands of Gy, while valley doses remain up to 100 times lower [16]. Consequently, the dose distribution on the target area displays peaks and valleys with a considerably high peak-to-valley dose ratio (PVDR) and can be modified by microbeam width and center-to-center spacing.

Recently, compact synchrotron x-ray sources received more attention as they provide essential prerequisites for the translation of MRT into clinics while overcoming the limited access to synchrotron facilities [18]. Nowadays, microbeam radiation therapy (MRT) is still a preclinical treatment method in radiation oncology with the potential to substantially improve the therapeutic efficacy of radiotherapy [19,20]. Over the years, an enormous amount of research has been devoted to the dosimetric and biological studies of MRT for cancer therapy, establishing the efficacy of MRT regarding enhanced normal tissue sparing in multiple animal experiments [8].

This project investigated the combined effect of spatial and temporal dose fractionation on two lung cell lines. The combination of spatial and temporal fractionation received little attention within the scientific community in the past, and respective studies were only conducted in the last couple of years. Fernandez-Palomo et al. investigated in 2020 temporally fractionated MRT in C57Bl/6 mice, thereby demonstrating enhanced tumor control as well as prolonged survival in the three temporal fractionated MRT group compared to the single MRT irradiated mice [21].

However, the radiobiological aspects of this novel approach are still partly unclear and need further validation. Since temporal dose fractionation is nowadays state-of-the-art in conventional cancer treatment schemes, the objective of the present study was to investigate and to analyze the effects of temporally fractionated MRT and BB on the therapeutic index by analyzing clonogenic cell survival in vitro, the current gold standard in radiobiology. Our study was conducted to compare the feasibility of two different MRT irradiation schedules, one with a steady MRT field throughout the four fractions and one with a 45° rotation per fraction. It is assumed that the rotation may have an even more pronounced effect on the increase in normal tissue protection as well as enhanced tumor cell kill at the same time.

For the spatial fractionated radiation modalities, the concept of the equivalent uniform dose (EUD) was applied. The underlying concept defines the absorbed dose, which would be delivered by conventional homogenous RT, causing the same biological effect of clonogenic survival given as a spatially fractionated absorbed dose [22]. In order to compute the EUD, the established model of the linear quadratic model (LQM) is used and requires the parameter fitting of individual cell line-dependent factors like the α and β values as well as the physical dose distribution [23].

## 2. Materials and Methods

### 2.1. Cell Lines

Two established human cell lines, i.e., A549, human-derived epithelial non-small cell lung carcinoma, and MRC-5, human-derived normal lung fibroblast cells, were obtained from the ATCC (American Type Culture Collection, ATCC, Manassas, VA, USA). The A549 was cultured in Dulbecco’s Modified Eagle Medium–high glucose (DMEM high glucose, Sigma-Aldrich, St. Louis, MO, USA) and MRC-5 in Dulbecco’s Modified Eagle Medium (DMEM/F-12 (1:1)), American Type Culture Collection (ATCC, USA). Additionally, 10% heat-inactivated fetal bovine serum (FBS, Sigma-Aldrich, USA) as well as 1% penicillin/streptomycin [10 mg/mL] (P/S, Sigma-Aldrich, USA) was supplemented for both cell lines. All cells were incubated at 37 °C in a regulated environment of atmospheric air containing a 21% volume fraction of O_2_ and 5% CO_2_. Both cell lines were grown as an adherent monolayer and trypsinized every 2–3 days.

### 2.2. Cell Survival Assay

In 1956, Puck and Marcus described a cell culture technique to assess the colony formation ability of a single cell. Since then, the colony formation assay (CFA) has been the gold standard for determining the survival and growth capacity of cells after treatment, e.g., ionizing radiation. This assay is based on the capacity preservation of single cells to produce a large number of progenies after treatment, which could cause apoptosis due to reproductive death or as a result of chromosomal damage. A colony is defined when it consists of at least 50 cells, which represents seven to eight proliferations [24].

All CFAs were performed as post-plating. First, the Ibidi µ-Dish (µ-Dish 35 mm, low, Gräfelfing, Germany) was used to seed the cells for irradiation in a density of 45,000–60,000 cells per dish. The irradiations were performed in four fractions per week, each after an exact 24 h interval. Following the last fraction, the CFAs were seeded after incubating the cells for 30 min at 37 °C and 5% CO_2_ following the last fraction into 6-well plates (TPP AG, Trasadingen, Switzerland). For this, cells were washed inside the Ibidi µ-Dish with 1 mL of PBS (Dulbecco’s Phosphate-Buffered Saline, Sigma-Aldrich, USA), and 0.5 mL of Trypsin (Trypsin- EDTA Solution, Sigma-Aldrich, USA) was added for 5 min. Afterwards, 1 mL of the corresponding medium was added to neutralize the reaction, and the cell suspension was transferred into a 15 mL centrifuge tube (VWR, Radnor, PA, USA). Each suspension was counted four times with a Neubauer chamber, and the corresponding number of cells were seeded into the 6-well plates. To enhance statistical accuracy and reproducibility, all experiments were performed with three biological replicates, each comprising six technical replicates.

The cells were allowed to incubate for fourteen days each. Afterward, the plates were washed with PBS and fixed with ice-cold methanol (Methanol ≥ 99.5% Ph. Eur., reinst, Carl Roth GmbH und Co. KG., Karlsruhe, Germany). The colonies were stained with crystal violet (Crystal-Violet, TUM University Hospital, Munich, Germany) and analyzed with the GelCount™ (GelCount™, Oxford Optronix, Abingdon, UK). A colony was counted if it consisted of at least 50 cells. Furthermore, the plating efficiency (PE) and survival fraction were determined. Plating efficiency is the percentage of cells with the ability to grow into colonies [24]:

(1) $$PE\;\left[\%\right]=\;\left(\frac{\left[No.\;colonies\;counted\right]}{\left[No.\;cells\;seeded\right]}\right)\cdot 100$$

Survival fraction (SF) is the ratio of colonies that arise after treatment with ionizing radiation, expressed in respect to the (PE) [24]:

(2) $$SF\;\left[\%\right]=\;\frac{\left[No.\;colonies\;counted\right]}{\left[No.\;cells\;seeded\right]\cdot PE}$$

### 2.3. Irradiation Procedures

Conventional irradiations were conducted using the RS225 (Xstrahl, Camberley, UK) with 220 kV, 10 mA, and a 0.15 mm copper filter. In order to generate a microbeam field, a bespoke multi-slit collimator was employed, which was described before [17]. The 7 mm thick tungsten multi-slit collimator mounted inside the small animal irradiator platform XenX (Xstrahl, UK) produced a 2 × 2 cm^2^ microbeam field. The platform accommodates a Comet MXR-225/22 x-ray tube (Varian Medical Systems, Palo Alto, CA, USA) installed on a rotating gantry. Irradiations were performed with 225 kV, 13 mA, a 0.15 mm copper filter, a slit width of 30 µm, a PVDR of 45.77 and a peak dose rate of 4.31 Gy/min, and the valley dose rate was 0.09 Gy/min. Film dosimetry was performed according to previously established protocols [25,26]. Films were scanned with a Reflecta ProScan 10T (Reflecta GmbH, Eutingen, Germany) diapositive scanner.

The following three treatment groups were investigated:

(1) BB_fx4_: Conventional broad-beam irradiation (BB) with 4 fractions.
(2) MRT_fx4_: 4 consecutive MRT irradiation with precise alignment of the microbeams (0°).
(3) MRT_fx4_ + R: 4 consecutive MRT irradiation with beam rotation by 0°, 45°, 90°, 135° around the beam axis.

To ensure a reproducible experimental setting with high quality, a U-shaped plastic stencil with markings was used for an exact positioning of the cell dishes at both microbeam modalities. The Ibidi µ-Dish, 35 mm, low, was repeatedly positioned at 0° for MRT irradiation without rotation for every fraction. The MRT irradiation with rotation was performed at the following angles: 0°, 45°, 90°, and 135°.

**Figure 1:**
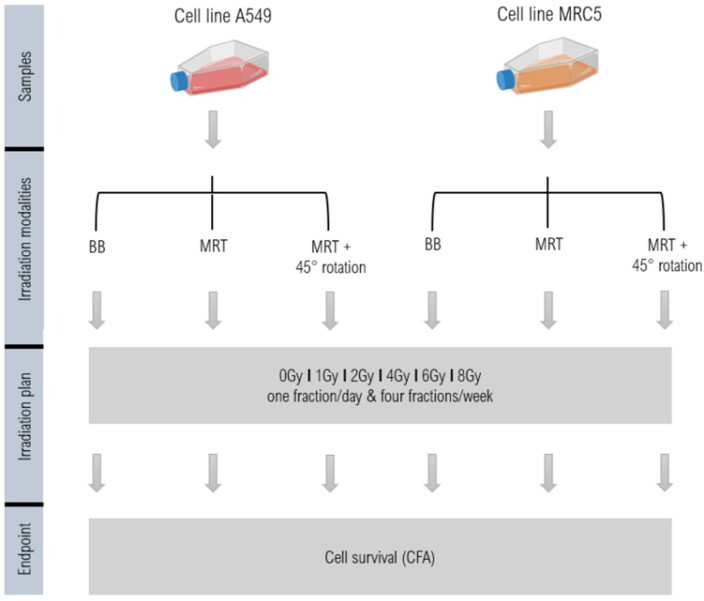
Schematic description of the workflow. Cells are seeded for the different treatment regimes in an Ibidi µ-Dish and irradiated with either BB, MRT or MRT with a rotation of 45° per fraction (MRT + R) over four consecutive days. Afterwards, the colony formation assay (CFA) is performed. The illustration was created with BioRender (https://www.biorender.com/).

A schematic overview of the workflow is given in Figure 1.

The fraction sizes were calculated using the linear quadratic model (LQM) and assuming the equal survival of cells. If cells are growing at a uniform density on the cell culture disk area *A*, the cell survival after a radiation treatment with N fractions can be calculated by the following:

(3) $${SF}_{fractionated}=\int_{x,y\epsilon A}\exp\left(-\sum_{i=0}^{N}\alpha D_{i}\left(x,y\right)+\beta {\left(D_{i}\left(x,y\right)\right)}^{\left(2\right)}\right)dxdy$$

Each fraction *i* has the dose distribution $D_{i}$(*x*, *y*). The equivalent uniform dose single fraction (${EUD}_{single\_fraction}$) [27] is the BB dose that would lead in a single fraction to the same survival, i.e.,

(4) $${SF}_{fractionated\;}=\;\exp\left(-\alpha \cdot {EUD}_{single\_fraction}-\beta \cdot {{EUD}_{single\_fraction}}^{2}\right)$$

Note that MRT + R will lead to a higher ${EUD}_{single\_fraction}$ than the overlapping MRT, when treating with the same fraction dose distribution. The LQM parameters *α* and *β* for both cell lines can be found in Table 1.

### 2.4. Statistical Analysis

Significance was assessed by an analysis of covariance (ANCOVA), which blends regression and analysis of variance (ANOVA), using Python (Version 3.11.10, Python Software Foundation, Wilmington, NC, USA). A *p*-value of 0.05 indicates that the difference between groups is statistically significant.

## 3. Results

### 3.1. Cell Survival

Using the results from the clonogenic cell survival assay, the effects of different temporally fractionated irradiation modalities of conventional broad-beam (BB) irradiation, overlapping microbeam radiation therapy (MRT), as well as MRT with a rotation of 45° for each fraction (MRT + R) on the cell lines A549 and MRC-5 were analyzed.

**Figure 2:**
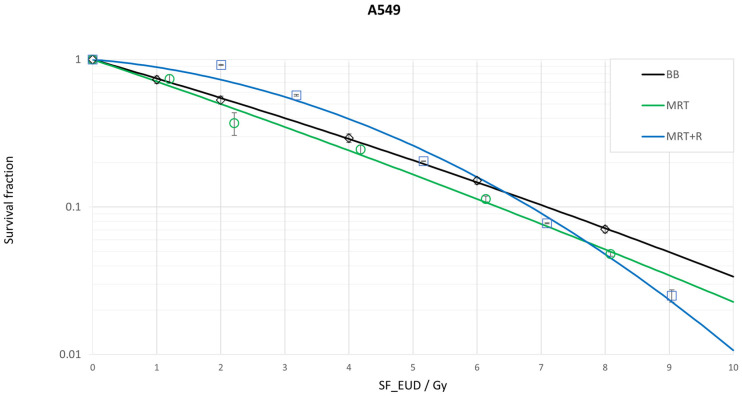
Cell survival curves for the lung tumor cell line A549 following three fractionated irradiation procedures: broad beam (BB), microbeam (MRT) and microbeam with a 45° rotation (MRT + R). In total, four temporally fractionation irradiations with a 24 h time interval between each fraction is performed. The corresponding survival fraction is plotted over an equivalent uniform dose single fraction.

For the lung tumor cell line A549, there was a statistically significant increase in cell survival with MRT + R compared to the overlapping MRT (*p* = 0.032) for doses below 7.7 Gy, as shown in Figure 2. Notably, the MRT + R curve crosses the BB cell survival curve at 6.5 Gy and the overlapping MRT at 7.7 Gy. Moreover, MRT + R demonstrates the lowest cell survival at 9.0 Gy and shows the steepest slope compared to MRT and BB.

However, no significant differences were observed between MRT + R and BB (*p* = 0.068) or between overlapping MRT and BB (*p* = 0.124) (see Table 2).

**Figure 3:**
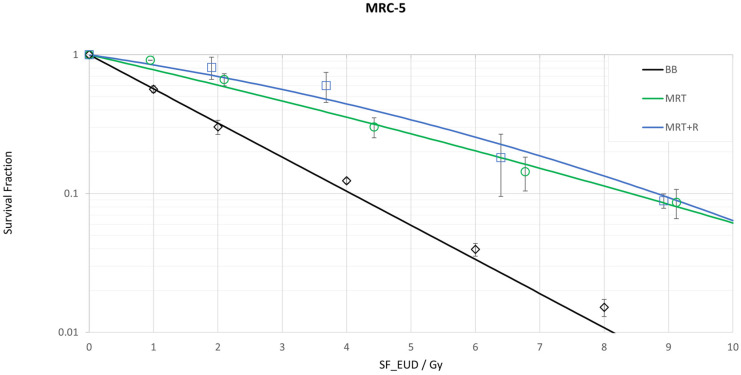
Cell survival curves for the normal lung cell line MRC-5 following three fractionated irradiation procedures: broad beam (BB), microbeam (MRT) and microbeam with a 45° rotation (MRT + R). Four temporally fractions are applied with 24 h between each fraction. The corresponding survival fraction is plotted over the equivalent uniform dose single fraction (${EUD}_{single\_fraction})$.

In the case of the normal lung cell line MRC-5, cell survival was significantly higher for both MRT modalities compared to the BB (*p* ≤ 0.01) (see Table 3). There was no significant difference (*p* = 0.153) in clonogenic cell survival between the two MRT modalities (overlapping MRT and MRT + R) within the MRC-5 cell line, as illustrated in Figure 3.

### 3.2. Alignment Precision

**Figure 4:**
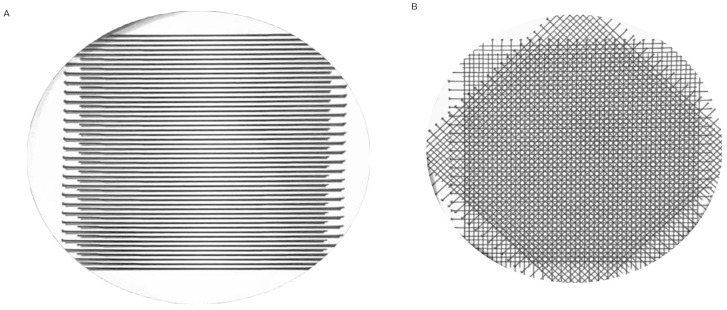
Dose profiles after film irradiation of Ibidi μ-Dishes with four dose fractions: (**A**) Dose profile following fractionated MRT irradiation. (**B**) Dose profile following fractionated MRT + R irradiation, by rotating the cell dish 45° per fraction.

To ensure precise and sharp dose-profile patterns of the fractionated MRT irradiations during the experiments, a radiochromic film was used for each experimental setting. Furthermore, to reproduce the accuracy of the positioning, the experiments were carried out with an EBT3 film attached to the Ibidi μ-Dish. The resulting images can be seen in Figure 4. Figure 4A shows the dose profile of the overlapping MRT fractions without rotation, and Figure 4B displays the microbeam dose profile of the MRT + R modality. The emerging pattern is the result of the 45° rotation at each fraction. Moreover, the corresponding dose-volume histogram can be seen in the Supplementary Data S1.

## 4. Discussion

The present investigation analyzed the effects of combining temporal and spatial fractionation on clonogenic cell survival in an in vitro setting. The data of clonogenic cell survival were collected for a non-cancerous normal lung tissue cell line and an NSCLC tumor cell line using the colony formation assay. The results demonstrate that the response of the investigated normal tissue cell line MRC-5 shows substantially increased survival for the spatial fractionated irradiation scheme. The data provide the first in vitro evidence that temporal fractionation in combination with microbeam irradiations leads to enhanced clonogenic cell survival in healthy MRC-5 cells when compared with conventional fractionated broad-beam irradiation. The LQM allows the comparison of spatially and temporal fractionated radiation schemes. Assuming that cells do not migrate between temporal fractions and react individually to radiation, the survival should be equal for all three studied schemes. Differences indicate that intercellular communication modifies cell survival.

For the investigated lung tumor cell line A549, the various radiation schemes show significant differences for the MRT with rotation (MRT + R) compared to the overlapping MRT but not in comparison to the BB fractionation. However, the MRT + R curve demonstrates the steepest survival curve and crosses the BB and the overlapping MRT curve at 6.5 Gy and 7.7 Gy, respectively. The findings show that overlapping MRT has beneficial outcomes on cell survival below 7.7 Gy when compared to MRT with rotation. At higher doses, the MRT + R demonstrates a decrease in cell survival. However, comparing the BB fractionation with the overlapping MRT, no difference was found for the tumor cell line, indicating that the EUD concept holds true in the setup used in this study for this modality. In standard MRT, cells in the valley regions receive little or no radiation, allowing for partial repair and survival. In MRT + R, as the beam rotates, new peaks of radiation overlap with previously spared valley regions, reducing the protective effect of dose heterogeneity. This leads to more widespread DNA damage across the entire cell population, increasing cell death at doses above 8 Gy.

Remarkably, the normal tissue sparing effect is found in our analysis. The normal tissue cell line MRC-5 demonstrates superior effects by highly statistically increased cell survival compared to the BB fractionation regime, independent from the irradiation angle of the MRT. No differences in cell survival were observed in the MRT treatment regimens with and without rotation. Therefore, we conclude that the clonogenic cell survival might be independent of the angle of the irradiation in this study.

In MRT, normal tissues display a higher apparent α/β ratio compared to conventional radiotherapy due to the distinct spatial fractionation and dose distribution. MRT administers extremely high doses in narrow microbeam peaks while leaving adjacent tissues in low-dose valleys, which allows normal cells to survive and efficiently repair damage. The β (dose-squared) component of the linear quadratic (LQ) model, representing sub-lethal damage repair, becomes less significant at ultra-high doses, making the damage response more linear and effectively increasing the α/β ratio. Furthermore, intercellular communication and cell migration enhance normal tissue resilience, further simulating a high α/β response.

In our current study, both cell lines exhibited a high α/β ratio. MRC-5 cells, which are fibroblasts, exhibit moderate proliferative activity and can show a higher α/β ratio than expected for normal tissues when exposed to radiation. Despite being normal cells, MRC-5 fibroblasts can exhibit a relatively strong initial radiation damage response (high α-component) similar to tumor cells when exposed to ionizing radiation. If cells experience incomplete or impaired DNA repair, the α/β ratio increases, indicating that damage accumulates in a more linear (α-dominant) manner rather than being repaired over time.

The findings in our study prove the healthy tissue sparing effect with spatial and temporal fractionated radiotherapy. Combining the results of both cell lines, a superior effect of MRT on healthy tissue sparing as well as equal tumor cell kill rates was demonstrated. Therefore, our data are well in line with the current literature, suggesting that temporally fractionated MRT leads to the same favorable tumoricidal effect as BB fractionated irradiations and reduced normal tissue damage [28,29,30]. Moreover, the healthy tissue sparing effect does not depend on precisely hitting the peaks in each treatment fraction, which would not be possible in future clinical studies. However, the concept of cross firing can be utilized in future clinical studies, which are technically easier and ensures higher reproducibility.

The enhanced survival of normal cells relative to cancer cells in microbeam radiation therapy (MRT) is a significant finding with profound biological and clinical implications. This differential survival indicates that MRT offers a higher therapeutic index, achieving greater tumor control with reduced damage to normal tissue. This advantage is particularly noteworthy compared to conventional radiotherapy, where normal tissue toxicity frequently constrains the maximum deliverable dose to the tumor.

Our data also demonstrate that the LQM and the EUD concept can be used to predict in vitro cell survival in temporal and spatial fractionation. It enables the calculation of treatment doses in complex temporal and spatially structured radiation treatments, consolidating previous findings without temporal fractionation. The rationale for delivering fractionated MRT from different angles assumed that this could enhance the therapeutic index even more [21,31].

More studies are needed to investigate the effect of fractionated MRT on different biological endpoints. The colony formation assay was chosen in the present study because it represents the gold standard in radiobiological research. Survival curves, which are generated in vitro, represent a powerful tool to compare and guide clinical response, marking them as the most vital biological assay in 2D preclinical radiooncology research. However, as only little is known about the biological effects of combining temporal and spatial dose fractionation, additional assays may provide important information that contributes to an expanded level of knowledge in this preclinical research field. The tumor cell line and normal tissue cell line chosen for this study originated from lung tissue and are well established for in vitro experiments. Additionally, only a few MRT experiments have been conducted with lung tumors so far [32], and, thus, the present study provides important information.

Apart from extending knowledge on temporally fractionated MRT through in vitro experiments, a transfer of these procedures will be required to perform in vivo experiments. Animal MRT experiments, mostly with rats, have been performed since the early nineties for various cancer sites and evaluated for a wide range of criteria (i.e., animal survival, tumor growth delay, tumor vasculature and oxygenation, bystander effects and immune response) [8]. The bystander effect in radiation therapy refers to the biological responses observed in non-irradiated cells as a result of signals received from nearby irradiated cells. In MRT, this phenomenon is particularly intriguing due to the extreme dose heterogeneity. Cells within the microbeam path receive very high radiation doses, while adjacent cells in the valley regions receive much lower or no direct radiation. Despite this, cells in the valley regions can exhibit DNA damage, apoptosis, or altered gene expression due to signals from directly irradiated cells. This response is mediated by factors such as reactive oxygen species (ROS), cytokines, and intercellular communication via gap junctions or extracellular vesicles [33]. These signals, including extracellular vesicles, initiate intercellular communication that can enhance DNA repair and tissue regeneration in normal cells, thereby contributing to the remarkable tissue-sparing effects observed in MRT.

However, animal models investigating the application of temporal dose fractionation in combination with spatially fractionated MRT are still scarce. Fernandez-Palomo et al. in 2020 conducted an experiment showing enhanced tumor control and prolonged animal survival [21]. Even though the feasibility of an in vivo study combining temporal and spatial fractionation was shown, future research will have to ascertain the veracity of the investigated parameters. Moreover, the authors argued that some issues regarding the technical framework of this study remain to be optimized, i.e., the peak dose per fraction, the number of fractions and planes of delivery, as well as the frequency of fractions [21].

Moreover, it is also very important to identify and validate biological mechanisms through which the beneficial outcomes of MRT can be explained. A review conducted by Moghaddasi et al. (2022) examines the radiobiological and treatment-related aspects of spatially fractionated radiotherapy (SFRT), including MRT, highlighting its ability to deliver highly focused radiation doses to tumors while sparing normal tissues [33]. The authors discuss how SFRT leverages differential dose distributions to induce cell death, explore bystander effects, and promote immune responses, all while minimizing collateral damage to healthy tissues. The review underscores the potential of SFRT to improve the therapeutic index, particularly through enhanced tumor control and reduced normal tissue toxicity, aligning with findings that MRT can achieve selective tumor kill with lower normal tissue damage.

The same holds true for fractionated MRT and the mechanisms behind its advantageous outcome concerning tumor control and normal tissue sparing [21]. First human trials of MRT are still pending, mostly because, up to date, MRT is only available at large synchrotron facilities. Nevertheless, a major step towards a clinical application of MRT could be realized by dividing the required high peak doses of the microbeam field into smaller fractions delivered in more than one treatment session. Hence, it might be the combination of temporal and spatial dose fractionation, which paves the way for clinical MRT applications in the future [21].

## 5. Conclusions

We performed experiments on a combination of spatial and temporal fractionation. The emerging picture from the data generated in this study suggests that tumor cells demonstrate equal sensitivity to temporally fractionated microbeam radiation as to conventional broad-beam irradiation. In contrast, normal tissue cells are able to achieve a superior survival when irradiated with a fractionated MRT scheme compared to BB radiation, which can widen the therapeutic window in future preclinical or clinical studies. We clearly demonstrated that a perfect alignment of microbeams in a temporal fractionation schedule is not necessary to create a tissue sparing effect in normal tissue cells. In tumor cells, the cell survival is the same for the conventional irradiated scheme as well as overlapping MRT investigated fractionation schemes when compared at equivalent doses calculated with the linear quadratic model. However, MRT combined with rotation demonstrated higher cell survival for doses up to 6.5 Gy.

The results from our study will support the clinical translation of MRT with the potential to open new chapters in modern radiotherapy, since microbeam radiotherapy offers a promising innovative treatment option in cancer treatment.

## Figures and Tables

**Table 1:** Alpha and beta values for A549 and MRC-5. Values are derived from uniform irradiated BB CFA with doses of 1, 2, 4, 6, 8 Gy. Computation is performed by using the LQM.

	α	β
A549	0.29075	0.01928
MRC-5	0.56606	0.00004

**Table 2:** Significance for the lung tumor cell line A549 survival fraction by using the ANCOVA test for the broad beam (BB), overlapping microbeam (MRT) as well as the microbeam with 45° rotation per fraction (MRT + R). *p*-Values of 0.05 are chosen to be statistically significant. *p* ≤ 0.05 *.

Significance A549 *p* ≤ 0.05 *	MRT	MRT + R	BB
MRT	-	0.032 *	0.068
MRT + R	0.032 *	-	0.124
BB	0.068	0.124	-

**Table 3:** Significance for the normal lung cell line MRC-5 survival fraction by using the ANCOVA test for the broad beam (BB), overlapping microbeam (MRT) as well as the microbeam with 45° rotation per fraction (MRT + R. *p*-Values of 0.05 are chosen to be statistically significant. *p* ≤ 0.05 *, *p* ≤ 0.001 **.

Significance MRC-5 *p* ≤ 0.05 *	MRT	MRT + R	BB
MRT	-	0.153	0.002 *
MRT + R	0.153	-	0.001 **
BB	0.002 *	0.001 **	-

## Data Availability

The raw data supporting the conclusions of this article will be made available by the authors on request.

## Supplementary Materials

The following supporting information can be downloaded at https://www.mdpi.com/article/10.3390/biomedicines13030678/s1, Supplementary Data S1: Dose-Volume Histogram for the microbeam irradiation setup.
