# Supplementary material for: The Combination of Temporal and Spatial Dose Fractionation in Microbeam Radiation Therapy
Source: Biomedicines. 2025 Mar 10;13(3):678. doi: 10.3390/biomedicines13030678 (PMC11940479; doi:10.3390/biomedicines13030678)
Supplement: Supplementary file 1 [file biomedicines-13-00678-s001.zip › biomedicines-3365673-supplementary.pdf]

Supplementary Data S1: Dose-Volume Histogram for the microbeam irradiation setup.

S1: Dose-volume histogram for the microbeam irradiation overlapping (blue) and rotation (orange)

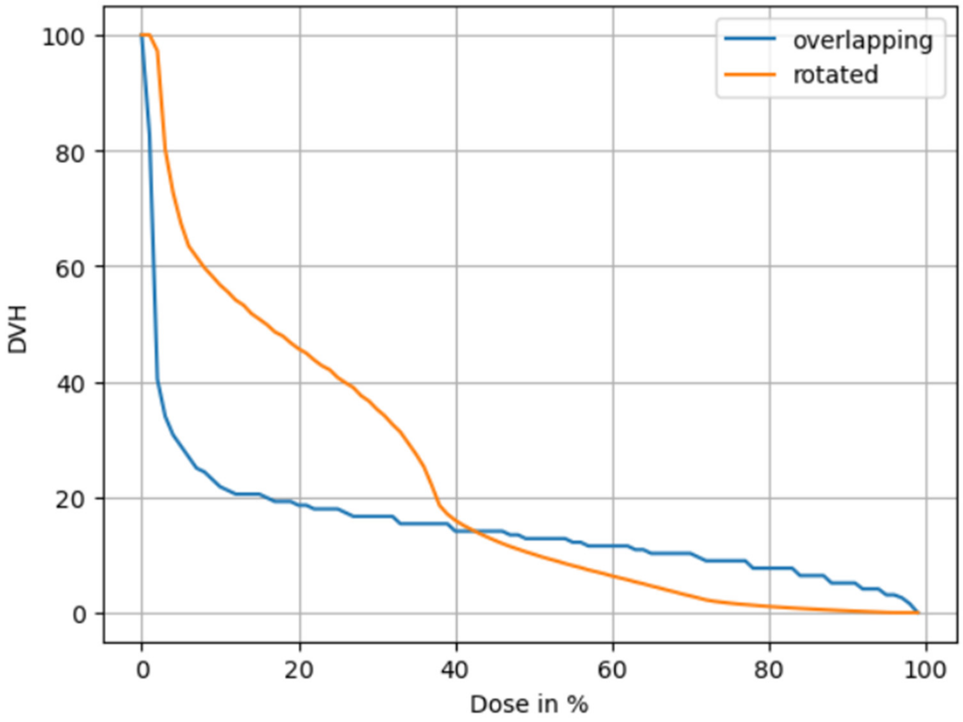

Supplementary Figure S1 Dose-volume-histogram for the microbeam irradiation overlapping (blue) and rotation (orange) in Dose [%].

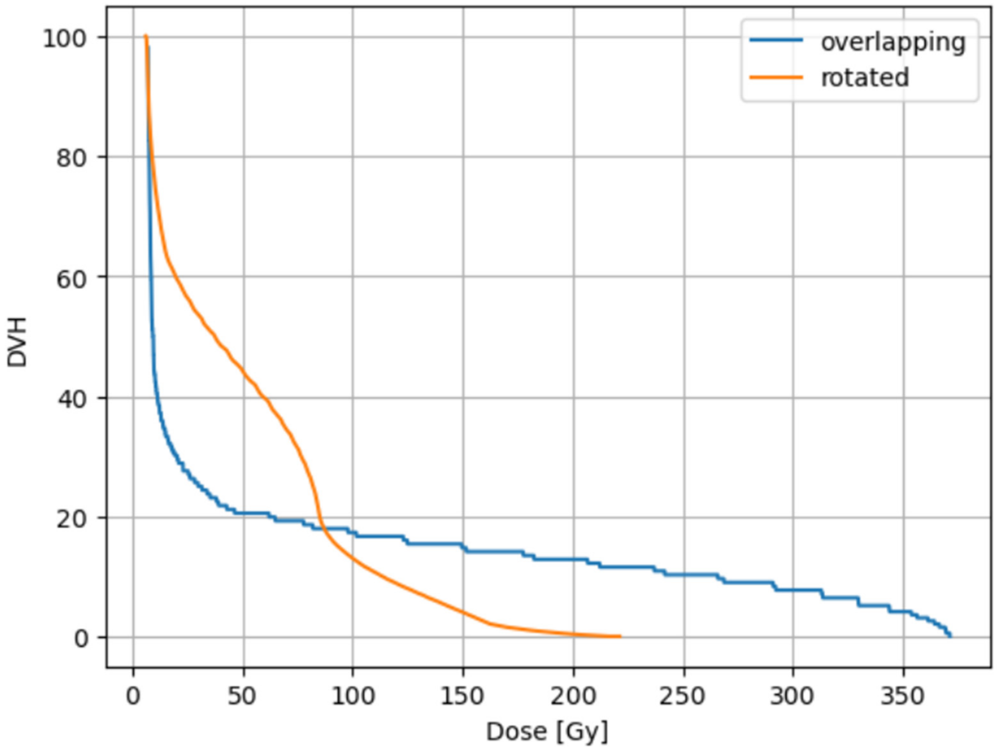

Supplementary Figure S2 Dose-volume-histogram for the microbeam irradiation overlapping (blue) and rotation (orange) in Dose [Gy].

The graph illustrates the dose-volume histogram (DVH) for a single exposure with either one microbeam field (blue) or a combination of four rotated microbeam fields (orange), as shown in Figure 3 A and B. The dose profiles were normalized to achieve an equivalent uniform dose (EUD) of 8 Gy for both scenarios. The graph indicates that the valley dose is the primary factor influencing survival according to the linear quadratic model, with values slightly below 8 Gy in both cases. In the blue DVH, the full width at half maximum (FWHM) of the beam is determined to be 12.8%. The center-to-center spacing between the beams is 402 µm, resulting in an FWHM of 51.5 µm. A broad beam penumbra is observed, attributed to scattering, the large source size, and the divergent nature of the beam.

For the rotated field crossing beam geometry, there are regions where 0, 1, 2, 3, and 4 beams overlap. According to statistical analysis, these areas occupy:

0 beams: 0.872^4 = 57.82%

1 beam: 4* 0.872^3*0.128 = 33.95%

2 beams: 6 * 0.872^2*0.128^2 = 7.47%

3 beams: 4 * 0.872*0.128^3 = 0,72%

4 beams: 0.128^4 = 0,03%

The regions with 0, 1, 2, and 3 beams can be observed in the histogram, while the 0.03% fraction with 4 beams is too small. Additionally, this fraction would result in a peak dose comparable to the single field peak dose. It is important to note that the fields were normalized to ensure equal survival according to the linear quadratic model. Peak regions are of minor importance for overall survival, as they are small and contribute minimally to the cohort of surviving cells.
